# Subtyping psychological distress in the population: a semi-parametric network approach

**DOI:** 10.1017/S204579601900026X

**Published:** 2019-05-15

**Authors:** S. de Vos, S. Patten, E. C. Wit, E. H. Bos, K. J. Wardenaar, P. de Jonge

**Affiliations:** 1 University of Groningen, University Medical Center Groningen, Interdisciplinary Center Psychopathology and Emotion regulation, Groningen, The Netherlands; 2 Departments of Community Health Sciences and Psychiatry, University of Calgary, Calgary, Alberta, Canada; 3 University of Groningen, Johann Bernoulli Institute of Mathematics and Computer Science, Groningen, The Netherlands; 4 Department of Developmental Psychology, University of Groningen, Faculty of Behavioural and Social Sciences, Groningen, The Netherlands

**Keywords:** Anxiety, data-driven, depression, *k*-means, network model, subtypes

## Abstract

**Aims:**

The mechanisms underlying both depressive and anxiety disorders remain poorly understood. One of the reasons for this is the lack of a valid, evidence-based system to classify persons into specific subtypes based on their depressive and/or anxiety symptomatology. In order to do this without *a priori* assumptions, non-parametric statistical methods seem the optimal choice. Moreover, to define subtypes according to their symptom profiles and inter-relations between symptoms, network models may be very useful. This study aimed to evaluate the potential usefulness of this approach.

**Methods:**

A large community sample from the Canadian general population (*N* = 254 443) was divided into data-driven clusters using non-parametric *k*-means clustering. Participants were clustered according to their (co)variation around the grand mean on each item of the Kessler Psychological Distress Scale (K10). Next, to evaluate cluster differences, semi-parametric network models were fitted in each cluster and node centrality indices and network density measures were compared.

**Results:**

A five-cluster model was obtained from the cluster analyses. Network density varied across clusters, and was highest for the cluster of people with the lowest K10 severity ratings. In three cluster networks, depressive symptoms (e.g. feeling depressed, restless, hopeless) had the highest centrality. In the remaining two clusters, symptom networks were characterised by a higher prominence of somatic symptoms (e.g. restlessness, nervousness).

**Conclusion:**

Finding data-driven subtypes based on psychological distress using non-parametric methods can be a fruitful approach, yielding clusters of persons that differ in illness severity as well as in the structure and strengths of inter-symptom relationships.

## Introduction

Internalising disorders such as depression and anxiety are among the most common mental illnesses and constitute a major burden on both patients and society (Baxter *et al*., 2014). In particular, major depressive disorder is projected to become the biggest contributor to the global burden of disease by 2030 (Day and Sweatt, 2012). Unfortunately, the mechanisms underlying both depression and anxiety disorders remain poorly understood. One of the reasons for this is that there is not yet a valid, evidence-based system to classify persons into more homogeneous subtypes based on their depressive and/or anxiety symptomatology. As a result, it has remained hard to account for the enormous variety in symptom profiles, comorbidity patterns, course trajectories and treatment effects that are observed across patients (Lamers *et al*., 2010; Strand *et al*., 2016). Several researchers have focused on better capturing and explaining this heterogeneity by the development of data-driven classification systems to subtype patients based on their depressive and/or anxiety symptomatology (e.g. Sullivan *et al*., 2002; Wanders *et al*., 2016).

Ideally, a symptom-based classification system should be usable to discern different types of individuals based on their clinical features, i.e. recognise the different symptom profiles that can occur. For example, a system should be able to reliably discern a patient with a psychosomatic symptom profile from a patient with mainly cognitive symptoms. Importantly, such a classification should be useable in research, but preferably also applicable in clinical settings (Epskamp *et al*., 2018). Ideally, new classifications should be developed using as few *a priori* assumptions about the nature of mental disorders as possible, since current classifications or labels have been shown to have limited validity (RE. 1989; Clark *et al*., 1995; Widiger and Clark, 2000; Kendell and Jablensky, 2003; Kraemer, 2007; Jablensky, 2016; Wakefield, 2016). Hence a model-free *non-parametric* approach to data-driven subtyping would be a suitable starting point. One of the best known non-parametric clustering methods is *k*-means clustering (Li *et al*., 2016). This method can be applied in a variety of ways. Given a sample of responses to a questionnaire, individuals can be clustered based on their raw item scores. Alternatively, one could take into account heterogeneity in the associations between the items, by computing a rough estimate of each person's sample covariance matrix with respect to the sample mean of each item. The unique elements of such a matrix would represent each person's variability with respect to the item sample means.

Suppose we have a non-parametrically derived cluster model of a dataset. How would we characterise and analyse the characteristics of the symptom patterns in the clusters? If symptomatology is to be an important aspect of a classification system for internalising problems, then it makes sense to use a methodology that reflects this emphasis on symptoms. The simplest approach would be to look at the patterns of symptom frequencies and compare these across the clusters. However, such an approach provides limited insight into the way the symptoms are inter-related within each cluster. One class of models that can be used to provide more insight into these inter-relationships are network models (Borsboom *et al*., 2011; Borsboom and Cramer, 2013). Network models have been used previously in a variety of psychiatry-related topics (e.g. Zdziarski and Simon, 2001; Cramer *et al*., 2010a; Wigman *et al*., 2013; Boschloo *et al*., 2015; Fried *et al*., 2017). In such models, symptoms are represented by nodes and the associations between symptoms are represented by edges (of varying strength) that connect the nodes. Network models have been used to analyse the network structure of the DSM (Boschloo *et al*., 2015), to study comorbidity (Cramer *et al*., 2010b), to differentiate between patient groups (Wigman *et al*., 2013) and to predict the prospective course of depression (Zdziarski and Simon, 2001). In the network approach, a mental disorder is assumed to be manifested as the result of the interplay among its constituent symptoms. This contrasts with the traditional view that symptoms reflect variations on a single latent construct that is responsible for all symptoms’ manifestations (Borsboom and Cramer, 2013). An advantage of the network approach is that one can use a large variety of mathematical tools to gain deeper insight into the network structure. For example, networks can be characterised by the comparative importance of nodes (a.k.a. node centrality) or by their global level of connectivity among nodes. Another possibility is to consider the distribution of a network's node centralities. It has been shown that networks in various real-life applications display a similar distribution of node centralities (Streicher *et al*., 2012). For example, networks whose node centralities follow a so-called power law have the property that around 20% of their nodes account for 80% of the total connectivity. Of course, other distributions are possible and can be investigated in the context of mental illness, where different (sub)types of patients may be characterised by different centrality distributions.

Given their advantages, networks seem to be a suitable tool for characterising and analysing data-driven clusters. However, some technical issues have to be overcome. One important difficulty with network analyses on self-reported symptom data is the fact that responses on items that assess symptoms can be highly skewed (e.g. suicidal ideation is a highly relevant symptom but only seldom reported), leading to non-normally distributed residuals and possibly biased results (e.g. Terluin *et al*., 2016). Fortunately, there are network modelling options that relax the assumption of normality and are less sensitive to the effects of skewed data distributions. One such model is the semi-parametric network model that uses a so-called *non-paranormal* distribution to estimate a network model on data, of which a transformation is normally distributed (Liu *et al*., 2012).

The goal of the present study is twofold. First, we aim to identify mental-disorder subtypes by using a non-parametric clustering method. To this end, we apply the *k*-means clustering algorithm to mental symptom data, collected in a large sample of household-dwelling adults in the Canadian Community Health Survey (CCHS), who were all assessed with the Kessler Psychological Distress Scale (K10). Because we are interested in inter-symptom relationships we apply the *k*-means algorithm to each person's matrix of item covariances with respect to the sample mean. Second, we aim to investigate the distinct characteristics of the identified clusters by using the above-described semi-parametric network models to evaluate cluster-specific symptom centrality, centrality distributions and overall network density.

## Methods

### Participants and procedures

The dataset came from the CCHS (Rumpf *et al*., 2018), which has an objective to gather health-related data from the Canadian general population on somatic disease and health conditions, lifestyle and social conditions as well as mental health and well-being. As part of the mental-health module, the K10 questionnaire was used to measure the depression and anxiety symptomatology. The mental health survey covers the population aged 15 years and over living in the ten Canadian provinces. Excluded from the survey's coverage are: persons living on reserves and other Aboriginal settlements; full-time members of the Canadian Forces and the institutionalised population. Altogether, these exclusions represent about 2% of the target population. From the CCHS data, we extracted the K10 items and considered only those observations, which had no missing data, resulting in a sample size of *N*  = 254 443.

### Measures

The K10 is a ten-item questionnaire with items rated on a 1–5 Likert scale that was designed to provide a measure of global psychological distress based on questions about anxiety and depressive symptoms that a person has experienced in the most recent 4-week period (Andrews and Slade, 2001; Furukawa *et al*., 2003). For an overview of the K10 items and their labels, see Appendix 1.

### Statistical analyses

This section describes how the clustering and subsequent network analyses were performed.

### *K*-means clustering

The general idea behind the first part of the analyses was to identify clusters based on how individuals varied around the grand mean responses on each K10 item. To do this, the responses on the items were first averaged across the whole sample, resulting in ten average scores. Next, we calculated an estimate of each person's variation around these averages by computing for each individual's response the quantities (*x*_*ij*_ – mean(*x*_*j*_))^2^. Here, *x*_*ij*_ denotes the response of person *i* to item *j*. These quantities, arranged as a matrix, form a rough estimation of a sample covariance matrix and serve as a measure of an individual's variability around the grand mean (Huggins, 2010). It is the upper off-diagonal elements of this matrix (i.e. the unique entries) that were used in the *k*-means cluster analysis. Each individual was represented by 45 entries (i.e. the number of unique entries in the matrix described above) instead of ten (the number of items in the used questionnaire), allowing for higher-resolution clustering. This way of clustering also takes into account the (potential) associations among the items. *K*-means clustering was performed using the *kmeans* function in the *R* standard library (‘stats’). Various cluster solutions were investigated, ranging from solutions with 2–8 clusters and the selection of the number of clusters was based on a balance between model fit and model parsimony.

### Network modelling

Having grouped people into clusters, a semi-parametric paranormal network model (Liu *et al*., 2012) was then fitted based on each cluster's raw symptom dataset. The main feature of the paranormal model is that it generalises the Gaussian network model by assuming that a certain transformation of the data is normally distributed instead of the data itself. In other words, this network model assumes that *f*(*X*) is normally distributed instead of *X* itself. This transformation *f* is non-parametrically estimated based on the data. This model is implemented in the *R* library ‘huge’ (Zhao *et al*., 2012). This approach differs from typical preprocessing techniques such as log-, squareroot or Box-Cox transformations where a specific parametric form of the transformation is assumed. Instead, the required transformation is estimated empirically from the data and then applied to each item. Running this model in each of the individual cluster datasets yielded the inverse of the sample covariance matrix (i.e. a precision matrix) for each cluster. We transformed this matrix into a matrix of partial correlation coefficients using a previously described transformation (Lauritzen, 1996).

### Analysis of network characteristics

#### Node centrality

The obtained partial correlation matrix was visualised as a network for each cluster, using R package ‘qgraph’ (Epskamp *et al*., 2012). For each network, we computed and compared various characteristics. Specifically, for each node in each network, we calculated the centrality (i.e. its importance in the network). There are various ways of defining node centrality in a network. We looked at node strength (the sum of absolute values of edges incident to a node), closeness (the average shortest path length between a node and all other nodes in a network) and betweenness (the number of times a node is on the shortest path between two other nodes; Harary, 2018). Afterwards, we investigated if the clusters differed with respect to their node centrality distributions using Kolmogorov–Smirnoff tests. This was done in a pairwise manner: distributions in two clusters were compared in each separate test.

#### Overall network density and connectivity

We computed and compared two global connectivity measures for each cluster's symptom network, see (Forbes *et al*., 2017). First, we computed the average of the absolute edge weights in each network to obtain a measure of network ‘density’. Second, we computed the ratio of realised edges to the total number of possible edges to obtain a measure of overall network ‘connectivity’. It is useful to consider both these measures because they provide different insights into global connectivity and need not necessarily be in agreement with each other (De Vos *et al*., 2017).

## Results

### *K*-means clustering

The decrease in the total sum of squares with each added cluster can be used to guide the selection of a model that balances sufficient explanation of heterogeneity with model parsimony. However, since the total sum of squares will keep decreasing with each added cluster, this selection will always require a certain measure of subjective judgement. In this study, a five-cluster model was selected as it represented a good compromise between model fit and model parsimony. Descriptive statistics for each cluster in this model are shown in Table 1. Cluster 1 contained the most people and had the lowest mean K10 score. This cluster seemed to represent (moderately) healthy people. Cluster 5 was the smallest cluster and had the highest mean K10 score. Clusters 2, 3 and 4 fell in-between in terms of size and mean sum scores and seemed to represent subgroups with moderate levels of psychopathology.

**Table 1.** Sample descriptive statistics per cluster

Cluster	1	2	3	4	5
Size	229 525	4550	5200	14 038	1130
Sex (%Female)	45.7%	34%	51.2%	36.7%	34.5%
Age group					
15–37 years	32.7%	36.4%	54.5%	37.8%	29.0%
37–59 years	34.8%	44.5%	31.5%	37.9%	51.6%
59–81 years	28.4%	17.2%	12.8%	20.8%	17.9%
K10 item score, mean (s.d.)					
Feeling tired for no good reason	1.85 (0.91)	3.84 (1.00)	2.65 (1.19)	3.34 (1.03)	4.34 (0.96)
Feeling nervous	1.74 (0.88)	3.74 (1.02)	2.60 (1.25)	3.03 (1.06)	4.43 (0.85)
Feeling so nervous that nothing could calm you down	1.09 (0.35)	2.62 (1.17)	1.60 (0.97)	1.80 (0.95)	3.74 (1.20)
Feeling hopeless	1.13 (0.41)	3.28 (0.93)	1.54 (0.83)	2.33 (0.96)	4.26 (0.85)
Feeling restless	1.51 (0.79)	3.22 (1.09)	4.26 (0.71)	2.39 (0.97)	4.23 (0.90)
Feeling so restless you could not sit still	1.15 (0.45)	2.61 (1.18)	3.91 (0.82)	1.68 (0.83)	3.79 (1.16)
Feeling depressed	1.48 (0.72)	3.85 (0.74)	2.10 (0.97)	3.05 (0.79)	4.54 (0.60)
Feeling so depressed that nothing could cheer me up	1.07 (0.29)	3.17 (0.98)	1.35 (0.66)	2.08 (0.97)	4.13 (0.87)
Feeling everything was an effort	1.37 (0.73)	3.84 (0.85)	2.16 (1.16)	3.21 (0.97)	4.49 (0.69)
Feeling worthless	1.09 (0.35)	3.17 (1.08)	1.36 (0.71)	2.19 (1.03)	4.18 (0.99)
K10 sum score groups (%)					
10–23	99.8%	0%	52.8%	27.2%	0%
23–37	0.2%	86.2%	47.2%	72.8%	0%
37–50	0%	13.8%	0%	0%	100%

### Network characteristics

**Fig. 1.**
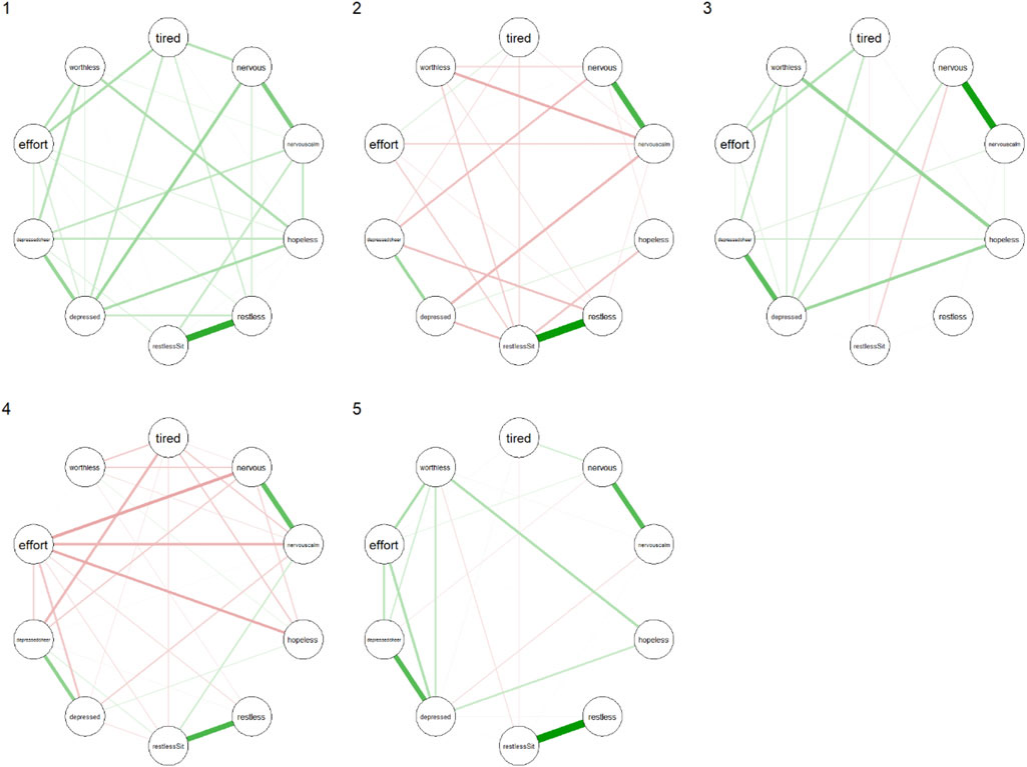
Partial correlation networks of clusters 1 through 5. For item node labels, see Appendix 1.

The partial correlation networks of the clusters are shown in Fig. 1. The networks of clusters 2 and 4 contained many negative edges while those of clusters 1, 3 and 5 contained mostly positive edges. Interestingly, the networks associated with clusters 1 and 4 had the largest global level of connectivity (see Table 2), regardless of the measure used. Even though cluster 1 contained the healthiest people and cluster 5 contained the people most afflicted with mental illness, the global connectivity measures showed a more densely connected network for cluster 1 than for cluster 5.

**Table 2.** Global connectivity measures according to two definitions of global connectivity

Global connectivity measure	Cluster				
	1	2	3	4	5
Connectivity	0.89	0.73	0.67	0.82	0.67
Density	0.073	0.059	0.052	0.067	0.055

**Fig. 2.**
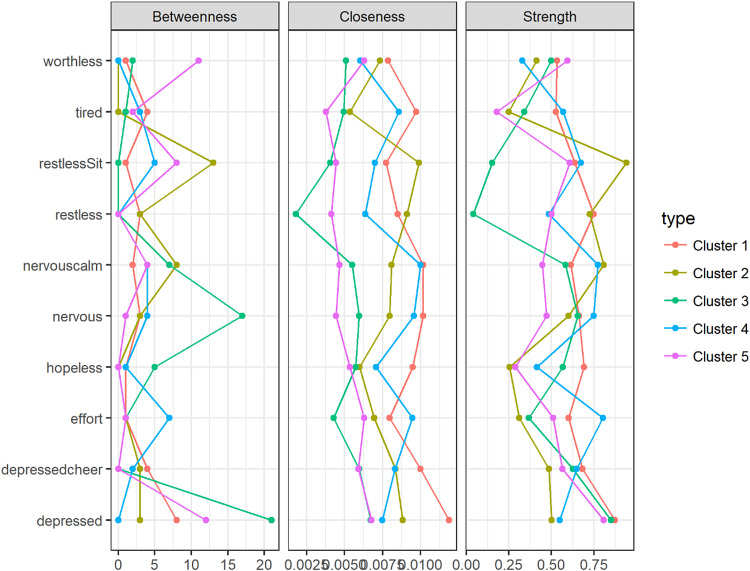
Centrality plot for clusters 1 through 5 for three centrality measures.

Node centrality measures are shown in Fig. 2. For each cluster, the nodes with the highest centrality are presented in Table 3. The symptom ‘Feeling depressed’ was the most prominent in clusters 1, 3 and 5 for all centrality measures. For cluster 5, the symptom ‘Feeling worthless’ made an entrance in each top 3. Cluster 3's most relevant nodes were all related to feeling depressed or nervous. For cluster 4, the symptoms ‘Feeling everything was an effort’, ‘Feeling nervous’ and ‘Feeling so nervous that nothing could calm you down’ comprised the top 3 most relevant nodes for all measures. Cluster 2 seemed to be dominated by ‘Feeling restless’ and ‘Feeling so restless you could not sit still’. Kolmogorov–Smirnoff tests did not indicate any statistically significant differences between the clusters' node centrality distributions.

**Table 3.** The three most central nodes per cluster, for each centrality measure

	Cluster				
Centrality measure	1	2	3	4	5
Strength					
1	Depressed	So restless you could not sit still	Depressed	Effort	Depressed
2	Restless	So nervous that nothing could calm you down	Nervous	So nervous that nothing could calm you down	So restless you could not sit still
3	Hopeless	Restless	So depressed that nothing could cheer me up	Nervous	Worthless
Closeness					
1	Depressed	So restless you could not sit still	Depressed	So nervous that nothing could calm you down	Depressed
2	Nervous	Restless	Nervous	Nervous	Effort
3	So nervous that nothing could calm you down	Depressed	So depressed that nothing could cheer me up	Effort	Worthless
Betweenness					
1	Depressed	Restless	Depressed	Effort	Depressed
2	Tired	So nervous that nothing could calm you down	Nervous	So restless you could not sit still	Worthless
3	So depressed that nothing could cheer me up	Nervous	So nervous that nothing could calm you down	Nervous	So restless you could not sit still

## Discussion

In this paper, we looked at non-parametric clustering of a large sample of individuals from the general Canadian population and applied a semi-parametric network model to each of these clusters, taking into account the skewness of the ordinal symptom reports often encountered in psychiatric questionnaire data from general population samples.

Our *k*-means clustering method was based on covariances instead of raw item scores or severity measures, yielding clusters not only differing clearly in levels of symptomatology but also in network structure. The analysis divided the dataset into five data-driven subgroups of people with varying levels of psychopathology. The cluster of people with the lowest symptom severity was by far the largest, in line with the fact that most people in the general population do not suffer from moderate to severe levels of mental illness, as measured by the K10. The other clusters seemed to represent groups of people with moderate (clusters 2, 3, 4) and high (cluster 5) levels of psychopathology.

The symptom networks showed pronounced differences across clusters, indicating substantial variation in symptom interdependency between clusters. Networks of clusters 2 and 4 differed from the other networks in that they contained mostly edges with negative weights. Another surprising difference was found in the networks of clusters 1 and 5. These clusters contained people with, respectively, the highest and the lowest symptom overall severity levels, yet the network of cluster 5 was less dense than the network of cluster 1. This seems to contradict earlier results in network modelling that suggest network density is positively associated with severity levels of psychopathology (Wigman *et al*., 2013, 2015; Boschloo *et al*., 2015; Pe *et al*., 2015; Van Borkulo *et al*., 2015; Borsboom, 2017). One possible reason for this discrepancy may be differences in methodology. For example, most of these previous studies did not account for the fact that psychopathology data often show substantial floor effects, especially in the subgroups with the lowest severity levels. This may result in lower variances in these groups, which may in part explain why observed connectivity measures may be higher in subgroups with higher symptom severity if such non-normal distributions are not accounted for (Terluin *et al*., 2016). Also, other methodological differences, for example, methods used for the detrending and centring of the data, have previously been shown to lead to differences in results (De Vos *et al*., 2017). Therefore, we opted for a non-parametric method in order to make no *a priori* assumptions about the underlying marginal distribution of the symptoms. Alternatively, it may simply not be true that a higher level of psychopathology implies a more highly connected network. In order to resolve this issue, more research is needed and, potentially, new theories should be developed in order to generate new testable hypotheses that can help the field to progress further. For instance, perhaps other network characteristics than connectivity could be more robustly associated with illness severity.

Inspection of the centrality indices can provide some insight into the relative importance of symptoms in a network. In directed networks, central nodes are often interpreted as nodes that play an important role in connecting other nodes in the network to each other. In cross-sectional, undirected networks, node centrality cannot be interpreted as nodes along directed paths. Here, high centrality of a node can be interpreted as an indication of a high rate of pairwise co-occurrences with its neighbouring nodes. *Vice versa*, a node that corresponds to a symptom that only shows little pairwise co-occurrences with other symptoms will have a comparatively low centrality in the network. Considering the most central nodes for each cluster's network, we saw that for people in clusters 1, 3 and 5, affect-related symptoms such as ‘Feeling depressed’ and ‘Feeling so depressed that nothing could cheer me up’ were most central. In cluster 5, the cognitive symptom of ‘Feeling worthless’ also stood out, indicating the importance of this symptom for people with severe psychopathology. In clusters 2 and 4, symptoms related to agitation (‘Feeling nervous’, ‘Feeling restless’) and energy (‘Feeling everything was an effort’) seemed to be most central. Importantly, these findings are relatively robust with respect to the choice of centrality measure. This indicates that, although far from complete, it seems possible to detect ‘from the ground up’ meaningful subgroups of individuals in the population, based on the structure of networks of depressive symptoms. The Kolmogorov–Smirnoff tests that were used to compare centrality distributions across clusters did not show significant differences in node centrality between clusters, but this may be partially explained by low sample size, since there are ten node centrality coefficients per network and the Kolmogorov–Smirnoff test is rather conservative.

Some limitations of this study need to be addressed. First, a problem with *k*-means clustering is that it is not always clear how to determine the optimal number of clusters, adding a subjective aspect to model selection. Future research might benefit from using more specialised approaches to model selection in a cluster context. For example, some methodologies might be borrowed from other statistical research areas, such as presented in Ceulemans and Kiers (2006, 2009). Second, this study was based on cross-sectional data, as a result of which we cannot say anything about symptom associations over time within persons. Finally, the K10 is dissimilar from other depression questionnaires, in that it contains items on depressive as well as anxiety symptoms. Future research is needed to investigate whether the results of this study can be replicated when using different samples and/or depression questionnaires.

In conclusion, using relatively simple techniques we were able to find clusters of people, which did not only differ in terms of symptom severity but also in terms of patterns of between-symptom associations, demonstrating a promising approach that can be used and further explored in future data-driven psychopathology subtyping studies.

## Availability of data and materials

The R scripts used to perform the analyses and generate the figures are available from the corresponding author upon request. The dataset is hosted by Statistics Canada under the Statistics Act, meaning that access to data might be available after following appropriate additional procedures. For more information, contact the corresponding author.

## Appendix A

**Appendix 1.** K10 item labels

Item number	Label	Label abbreviated
1	Feeling tired for no good reason	Tired
2	Feeling nervous	Nervous
3	Feeling so nervous that nothing could calm you down	NervousCalm
4	Feeling hopeless	Hopeless
5	Feeling restless	Restless
6	Feeling so restless you could not sit still	RestlessSit
7	Feeling depressed	Depressed
8	Feeling so depressed that nothing could cheer me up	DepressedCheer
9	Feeling everything was an effort	Effort
10	Feeling worthless	Worthless
